# Comparative genomic characterization of multidrug-resistant *Citrobacter* spp. strains in Fennec fox imported to China

**DOI:** 10.1186/s13099-021-00458-w

**Published:** 2021-10-13

**Authors:** Jie Qin, Yishu Zhao, Aifang Wang, Xiaohui Chi, Peipei Wen, Shuang Li, Lingjiao Wu, Sheng Bi, Hao Xu

**Affiliations:** 1 Emergency Department of Taizhou Hospital, Taizhou, China; 2 Department of Rheumatology and Immunology, Shandong Provincial Hospital, Jinan, China; 3 Department of Laboratory Medicine, Zhucheng People’s Hospital, Zhucheng, China; 4 Collaborative Innovation Center for Diagnosis and Treatment of Infectious Diseases, State Key Laboratory for Diagnosis and Treatment of Infectious Diseases, The First Affiliated Hospital, School of Medicine, Zhejiang University, Hangzhou, China

**Keywords:** *Citrobacter freundii*, Fennec fox, Comparative genomic analysis, *Bla*_CTX-M-13_, *qnrS1*

## Abstract

**Background:**

To investigate the antimicrobial profiles and genomic characteristics of MDR-*Citrobacter* spp. strains isolated from Fennec fox imported from Sudan to China.

**Methods:**

Four *Citrobacter* spp. strains were isolated from stool samples. Individual fresh stool samples were collected and subsequently diluted in phosphate buffered saline as described previously. The diluted fecal samples were plated on MacConkey agar supplemented with 1 mg/l cefotaxime and incubated for 20 h at 37 °C. Matrix-assisted laser desorption/ionization–time of flight mass spectrometry (MALDI–TOF–MS) was used for identification. Antimicrobial susceptibility testing was performed using the broth microdilution method. Whole-genome sequencing was performed on an Illumina Novaseq-6000 platform. Acquired antimicrobial resistance genes and plasmid replicons were detected using ResFinder 4.1 and PlasmidFinder 1.3, respectively. Comparative genomic analysis of 277 *Citrobacter* genomes was also performed.

**Results:**

Isolate FF141 was identified as *Citrobacter cronae* while isolate FF371, isolate FF414, and isolate FF423 were identified as *Citrobacter braakii.* Of these, three *C. braakii* isolates were further confirmed to be extended-spectrum β-lactamases (ESBL)-producer. All isolates are all multidrug resistance (MDR) with resistance to multiple antimicrobials. Plasmid of pKPC-CAV1321 belong to incompatibility (Inc) group. Comparative genomics analysis of *Citrobacter* isolates generated a large core-genome. Genetic diversity was observed in our bacterial collection, which clustered into five main clades. Human, environmental and animal *Citrobacter* isolates were distributed into five clusters.

**Conclusions:**

To our knowledge, this is the first investigation of MDR-*Citrobacter* from Fennec Fox. Our phenotypic and genomic data further underscore the threat of increased ESBL prevalence in wildlife and emphasize that increased effort should be committed to monitoring the potentially rapid dissemination of ESBL-producers with one health perspective.

**Supplementary Information:**

The online version contains supplementary material available at 10.1186/s13099-021-00458-w.

## Background

The worldwide increase and spread of infections caused by multidrug-resistant (MDR) Gram-negative bacteria of human and animal origin is a significant global public health burden in recent decades [1, 2]. Enterobacteriaceae are common bacteria and usually associated with different types of community- and hospital-acquired infections and sometimes even animal infections [3–6]. Thus, antimicrobial resistance (AMR) in these bacteria has significantly potential impacts on the control of AMR from the perspective of the One Health concept [7, 8]. Among these organisms, extended-spectrum β-lactamase (ESBL)-producing Enterobacteriaceae are recognized as the most prevalent group of pathogens due to their mobility [9, 10]. Treatment of infections caused by ESBL- and AmpC-producing Enterobacteriaceae strains is challenging, due to the emergence and spread of carbapenem resistance in ESBL-producing Enterobacteriaceae isolates, which is of particular clinical relevance [11, 12].

The ESBLs are usually carried by mobile genetic elements, such as a variety of self-transferring plasmids, which can be transferred to other bacteria [13–15]. Thus far, several ESBL types have been identified in Enterobacteriaceae isolates of which the CTX-M, TEM, and SHV β-lactamases are the most prevalent groups [16, 17]. It is noteworthy that ESBL- and carbapenemase-producing Enterobacteriaceae occurring in animals has become a public-health issue in recent years [4, 17]. Zoonotic pathogens contributed to the cross-transmissions of ESBL-producing Enterobacteriaceae [18]. Previously, we also reported the detection of the transmission of AMR across human, animals and environmental compartments in China [8, 19, 20].

*Citrobacter* spp. are common Gram-negative bacilli and widely found in water, food, soil, and intestines of animals and humans [21]. AMR encoding genes are frequently reported in *Citrobacter freundii*, and it became a reservoir of antibiotic resistance genes in recent years [22, 23]. In addition, MDR *C. freundii* has been reported in numerous hosts including but are not limited to humans [24–26]. In this work, we identified four MDR *Citrobacter* isolates in Fennec fox imported from Sudan to China. Antimicrobial susceptibility tests, conjugation experiments, whole-genome sequencing, and comparative genomic analysis were performed to study the molecular characteristics of these MDR strains.

## Results

### Isolation and identification

In this work, four *Citrobacter* spp. isolates resistant to cephalosporins were cultivated by selective medium plates. Among these strains, isolate FF141 was identified as *Citrobacter cronae* while isolate FF371, isolate FF414, and isolate FF423 were identified as *Citrobacter braakii* (Additional file 1: Figure S1). Of these, three *C. braakii* isolates were further confirmed to be ESBL-producer (Table 1).

**Table 1:** The minimum inhibitory concentration (MIC) of four *Citrobacter* spp. isolates

Isolate	TZP	CTX	CAZ	CPO	IPM	MEM	AMK	ATM	CIP	CHL	FLR	GEN	TOB	FOS	TGC	COL
FF141	64	32	64	1	0.25	0.0075	8	64	0.25	64	16	4	1	0.25	0.25	2
FF371	128	> 128	64	128	0.25	0.003	32	128	16	128	128	> 128	64	256	0.25	2
FF414	2	> 128	64	128	0.25	0.003	16	128	16	128	128	> 128	64	256	0.25	2
FF423	2	> 128	64	> 128	0.25	0.003	16	128	16	128	> 128	> 128	64	256	0.25	2

Concentration unit: μg/ml

*TZP* Piperacillin/tazobactam, *CTX* cefotaxime, *CAZ* ceftazidime, *CPO* cefpirome, *IMP* Imipenem, *MEM* meropenem, *AMK* amikacin, *ATM* aztreonam, *CIP* ciprofloxacin, *CHL* chloramphenicol, *FLR* florfenicol, *GEN* gentamicin, *TOB* tobramycin, *FOS* fosfomycin, *TGC* tigecycline, *COL* colistin

### Assessment of antibiotic susceptibility and MLST analysis

Four *Citrobacter* isolates are all MDR with resistance to multiple antimicrobials (Table 1). The total resistance rate was observed for cefotaxime, ceftazidime, aztreonam, and chloramphenicol (100%). All the isolates were susceptible to imipenem, meropenem, amikacin, tigecycline, and colistin. Interestingly, only FF371 was resistant to piperacillin-tazobactam. Among these *Citrobacter* isolates, we found two sequence types (STs), which were ST350 (FF371, FF414, and FF423) and ST370 (FF141).

### Resistant and virulence determinants of *Citrobacter* isolates

**Fig. 1:**
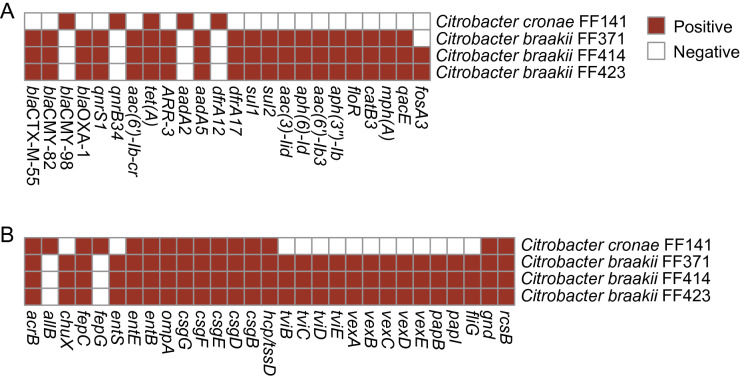
**A** Antimicrobial resistance gene profiles of 4 MDR-*Citrobacter* isolates. The heatmap is used to display the types of acquired AMR genes. Brown indicates the presence of AMR genes, whereas colorless correspond to the absence of the AMR genes. **B** The heatmap is used to display the types of virulence-associated genes. Brown indicates the presence of virulence genes, whereas colorless correspond to the absence of the virulence genes

The acquired resistance genes detected in four *Citrobacter* isolates are summarized in Fig. 1A. The following genes were identified in three *C. braakii* isolates: the phenicol resistance gene *floR*; the trimethoprim resistance gene *dfrA17*; the tetracycline resistance gene *tet(A)*; the disinfectant resistance gene *qaeE*; the quinolone resistance gene *qnrS1*; the fluoroquinolone and aminoglycoside resistance gene *aac(6ʹ)-Ib-cr*, the aminoglycoside resistance genes *aph(6)-Id*, *aac(6')-Ib3*, *aph(3ʺ)-Ib*, *aac(3)-IId*, and *aadA5*; the macrolide resistance gene *mph(A)*; the β-lactam resistance genes *bla*_CTX-M-55_, *bla*_CMY-82_, and *bla*_OXA-1_; the rifampicin resistance gene *ARR-3*; sulphonamide resistance genes *sul1*, and *sul2*. Of note, *C. braakii* FF414 and FF423 encoded the fosfomycin resistance gene *fosA3*, while *C. cronae* FF141 and *C. braakii* FF371 were negative*.* Among four isolates, FF141 encoded fewer resistance genes than other isolates, which carried *bla*_CMY-98_, *qnrB34, tet(A)*, *dfrA12*, and *aadA2.* Plasmids of incompatibility (Inc) group pKPC-CAV1321, was identified in FF141, and IncR was detected in other *Citrobacter braakii* isolates (Additional file 2: Table S1). The antimicrobial genes *dfrA12*, *tet(A)*, and *aadA2* genes were co-harbored by IncR plasmid in three *C. braakii* isolates.

Virulence gene analysis showed that the presence of genes encoding efflux pump protein (*arcB*), the transport of siderophores (*fepC*), enterobactin (*entB* and *entE*), outer membrane protein A (*ompA*), extracellular nucleation factors (*csgB*, *csgD*, *csgE*, and *csgF*), and type VI secretion system-related proteins (*hcp/tssD*) in all isolates (Fig. 1B). Moreover, three *C. braakii* isolates also carried genes that involved polymer synthesis (*tviB–tviE*), and cell surface localization of the CPS (*vexA–vexE*).

### Comparative genomics analysis of *Citrobacter* isolates

**Fig. 2:**
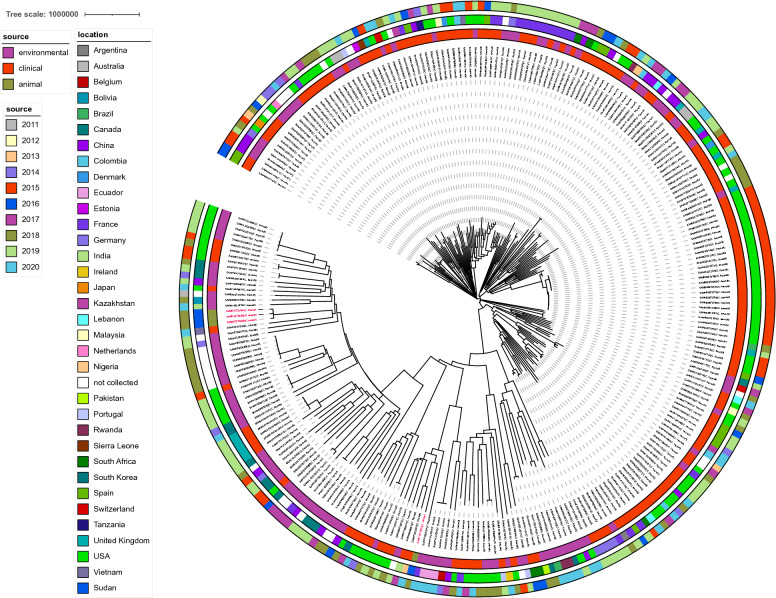
A core-genome analysis of 277 *Citrobacter* strains, including four isolates from this study and 273 strains downloaded from NCBI genome database. The year of the isolation is labelled in the outer ring. The source of the strains is presented in the middle ring. The location of the isolates is colored in the inner ring. Isolates identified in this study were colored in red. NA, details regarding the region of the strains is not available. The bar shows 100,000 nucleotide substitution per position

Roary matrix-based gene sequence analysis generated 60,923 total genes and a large core-genome of 1578 gene clusters of 277 whole genomes. The whole-genome phylogeny (Fig. 2) revealed a population structure. Genetic diversity was observed in our bacterial collection, which clustered into five main clades. Interestingly, *C. braakii* isolates have close relatedness with a clinical isolate (SAMD00112928, *C. freundii*) from Vietnam and an environmental strain (SAMN11928073, *C. freundii*), while *C. cronae* FF141 showed a high similarity with a clinical isolate from Nigeria (SAMN13830004, *C. freundii*) and a clinical isolate from India (SAMN06660612, *C. freundii*) (Fig. 2, Additional file 3: Table S2).

## Discussion

The wide dissemination of MDR Enterobacteriaceae is a global concern with one health perspective [27]. In clinical settings, *Citrobacter* spp. isolates represent up to 6% of all isolated Enterobacteriaceae from clinical specimens [28]. Members of the genus *Citrobacter* are reported to associate with nosocomial infections for a high mortality rate [29]. The *Citrobacter* genus is most closely related to *Escherichia coli* and *Salmonella*, and is divided into 11 different genomospecies [30]. There is some information indicating a high prevalence of MDR Enterobacteriaceae in wild animals [31, 32]. Although previous studies suggest that fur animals are potential reservoirs of AMR [5, 33], little is known about the antimicrobial patterns, and genomic characteristics of MDR *Citrobacter* isolates from wildlife. In the present study, we first described the isolation of MDR-*Citrobacter* strains from Fennec fox. We subsequently obtained the antimicrobial resistance profiles and genomic information by AST and WGS. We also identified antimicrobial resistance and virulence-associated genes.

*Citrobacter* spp. isolates can have chromosomal AmpC β-lactamases, as well as plasmid encoded carbapenemases, which results in ineffective of many antimicrobial agents for treatment [20, 22, 34, 35]. *C. cronae* was identified as a new *Citrobacter* species from human stool samples very recently [28], the antimicrobial profiles of *C. cronae* are largely unknow. In this study, we first identified an AmpC β-lactamase encoding gene (*bla*_CMY-98_) in *C. cronae*, which provides a glimpse of antimicrobial insight into this species. Occurrence of ESBL-producing *C. braakii* isolated from animals and food product are occasionally reported [36]. Our detection of three ESBL-producing *C. braakii* isolates from Fennec fox further suggests the risk of zoonotic potential MDR *C. braakii* from animals and animal products deservedly garners considerable attention.

Previous investigations found that CTX-M-55 became one of the prevalent ESBL type detected among clinical, animals, and environment in some countries [13, 14, 37, 38]. Very recently, the occurrences of CTX-M-55-producing *Escherichia coli* were also increasingly reported in environment and diverse animal species in Europe [39, 40]. Our previous work confirmed that CTX-M-55-producing *Escherichia coli* was the most prevalent ESBL-producer from Fennec fox [5]. These findings further strengthened that wildlife may act as potential reservoirs and vectors of CTX-M-55, although some of these isolates carried *bla*_CTX-M-55_ genes on the chromosome.

Interestingly, the diverging clonality of the human, environmental and animal *Citrobacter* isolates was confirmed by the fact that strains originating in these three sources distributed into five clusters. It is still not sure whether the ecological and animal strains are highly related to the human strains in terms of genetic phylogeny. The previous investigation highlighted the challenges associated with species designation of *Citrobacter* by core genome analysis, particularly in regards to *Citrobacter freundii*, which did not constitute a discrete phylogenetic group [41]. As we noted in our data, *C. cronae* and *C. braakii* strains were clustered into the same clade, which suggests further accurate taxonomic inquiry is needed to clarify the lineage of *Citrobacter* members.

## Conclusion

In summary, this investigation involved the first survey of MDR *Citrobacter* isolates in Fennec fox. We characterized the phenotypic characteristics and genomic basis of MDR *Citrobacter* strains. Fennec fox may serve as a common vector for the rapid dissemination of ESBL-encoding genes via animal contact and thereby threaten public health. Our findings further underscore the threat of increased ESBL prevalence in Enterobacteriaceae, and improved multisectoral surveillance for ESBL-producing *Citrobacter* is warranted.

## Methods and materials

### Bacterial identification and isolation of *Citrobacter* strains

We collected 168 stool samples of wild Fennec fox imported from North Africa to China [5]. Stool samples were cultured by MacConkey agar supplemented with 1 mg/l cefotaxime as described previously [5]. Bacterial identification was conducted by MALDI–TOF MS (Bruker, Bremen, Germany) as described [15]. Confirmation of ESBL-producing isolations was further performed by a standard double-disk diffusion method as defined by the Clinical and Laboratory Standards Institute (CLSI) (https://clsi.org/).

### Antimicrobial susceptibility testing (AST)

Susceptibility to 16 antibiotics (amikacin, aztreonam, cefpirome, cefotaxime, ceftazidime, chloramphenicol, ciprofloxacin, florfenicol, fosfomycin, gentamicin, imipenem, meropenem, piperacillin–tazobactam, polymyxin E, tigecycline, tobramycin, and trimethoprim–sulfamethoxazole) for four MDR *Citrobacter* isolates were evaluated. The MICs were determined via an agar dilution method for all antibiotics except for colistin and tigecycline, for which a broth microdilution method was used according to the CLSI standards. *E. coli* ATCC 25922 was used as control.

### Whole-genome sequencing (WGS) and bioinformatics analysis

Genomic DNA was extracted from four MDR *Citrobacter* isolates using the Qiagen Blood/Tissue kit (Qiagen, Hilden, Germany) [6]. The sequencing library was prepared by using Illumina Nextera XT kit (Illumina, San Diego, CA, USA) and sequenced using the Illumina NovaSeq 6000-PE150 platform (Illumina). Paired reads were then assembled into scaffolds using Velvet version 1.2.10 [42]. Acquired antimicrobial resistance genes and plasmid replicons were performed using the CGE server (http://www.genomicepidemiology.org). Antibiotics Resistance Genes (ARGs) were identified using the ResFinder 4.1 database [43]. Genotyping was performed to query the seven domesticated genes (*aspC, clpX, fadD, mdh, arcA, dnaG, and lysP*) via the MLST database (https://pubmlst.org/organisms/citrobacter-spp). We further created a core genome-based phylogenetic tree using 4 *Citrobacter* genomes sequenced in this study and 272 randomly selected publicly available *Citrobacter* genomes (Additional file 1: Table S1). The isolate collection includes strains from clinical (n = 159) and the environment (n = 133) sources that were widely distributed over time and geographical locations. *Citrobacter* genomes were annotated using Prokka [44] and RAST tool [45]. The core genes were identified using Prokka [44] and maximum likelihood-based phylogenetic reconstruction was performed with Roary [46]. Phylogenetic tree visualizations were generated by using iTOL (https://itol.embl.de/).

### Plasmid characterization

The transferability of plasmids carrying MDR encoding genes was determined by filter mating as described previously [19]. Animal isolates and *Escherichia coli* J53 were used as donors and acceptors, respectively. The animal isolates and J53 strains were mixed in (LB) broth at a ratio of 1:3 and incubated at 37 °C for 18 h. Transduction and binding were selected on MacConkey agar plates containing cefotaxime (2 μg/ml) and sodium azide (150 μg/ml) for 12 h. Susceptibility test was performed to determine the horizontal transferability of drug resistance, and the corresponding transduction conjugate was confirmed by PCR amplification and pulsed field gel electrophoresis (PFGE) (Additional file 2: Table S2).

## Supplementary Information

**Additional file 1: Figure S1.** MS fingerprinting spectrum for each of the four identified Citrobacter strains result of Matrix-assisted laser desorption/ionization-time of flight mass spectrometry (MALDI-TOF-MS).

**Additional file 2: Table S1.** List of information for the four genomes that were sequenced in this study.

**Additional file 3: Table S2.** List of strains and genome sequences that were used in this study.

## Data Availability

The whole-genome sequences of four *Citrobacter* spp. isolates have been deposited in the GenBank under the BioProject number PRJNA656097.
