# Supplementary figures and images for: Comparative genomic characterization of multidrug-resistant Citrobacter spp. strains in Fennec fox imported to China
Source: Gut Pathog. 2021 Oct 13;13:59. doi: 10.1186/s13099-021-00458-w (PMC8513245; doi:10.1186/s13099-021-00458-w)

A

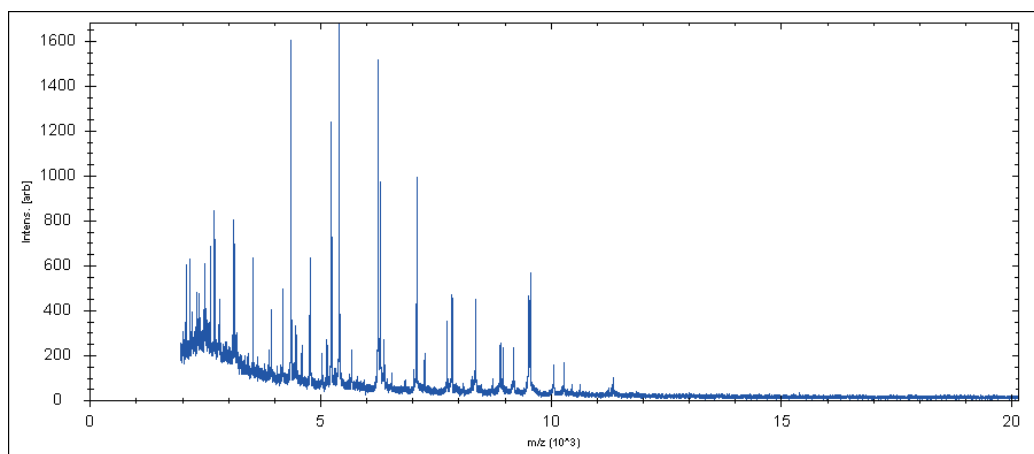FF371(*C. braakii*)

B

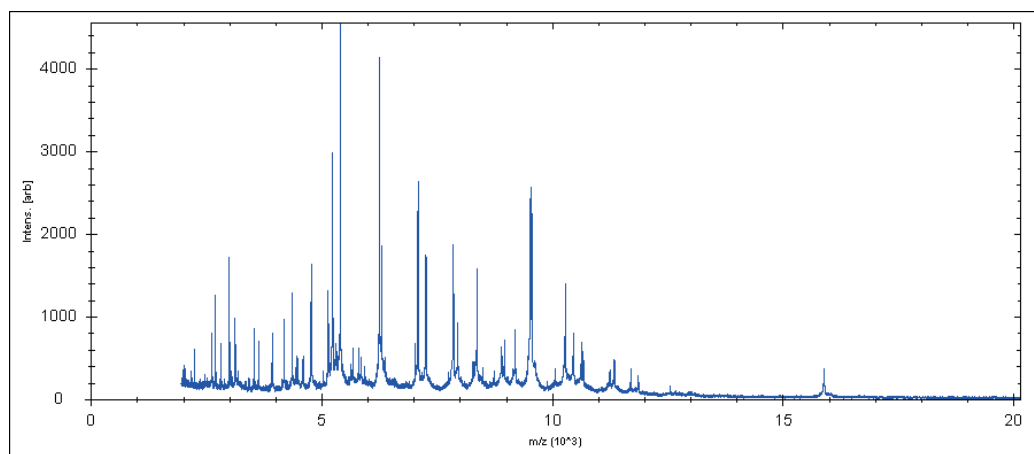FF414(*C. braakii*)

C

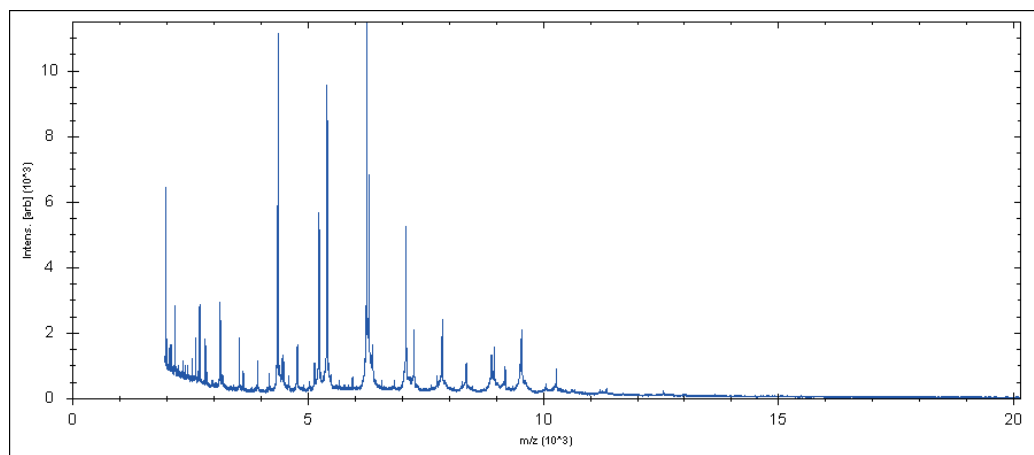FF423(*C. braakii*)

D

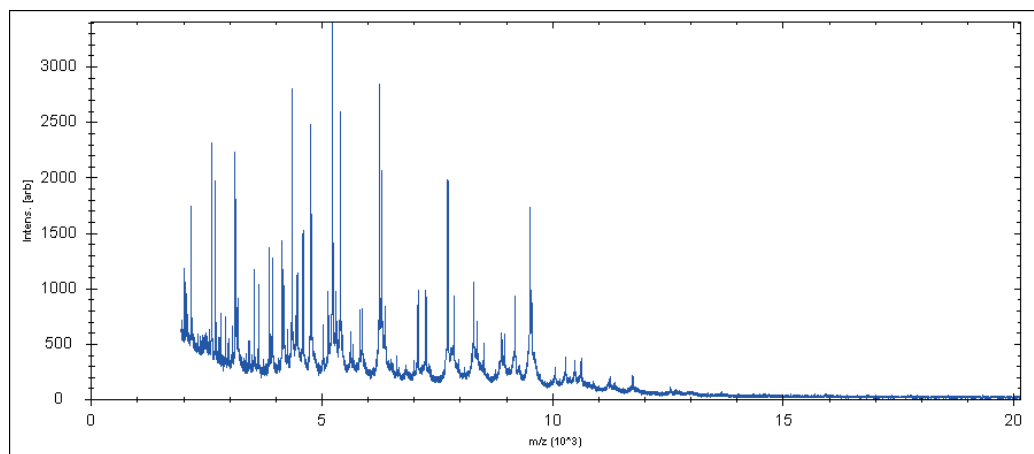FF141(*C. cronae*)

Supplement: Supplementary file 1 — Additional file 1: Figure S1. MS fingerprinting spectrum for each of the four identified Citrobacter strains result of Matrix-assisted laser desorption/ionization-time of flight mass spectrometry (MALDI-TOF-MS). [file 13099_2021_458_MOESM1_ESM.pdf]
