# Supplementary material for: Comparative genomic characterization of multidrug-resistant Citrobacter spp. strains in Fennec fox imported to China
Source: Gut Pathog. 2021 Oct 13;13:59. doi: 10.1186/s13099-021-00458-w (PMC8513245; doi:10.1186/s13099-021-00458-w)
Supplement: Supplementary file 2 — Additional file 2: Table S1. List of information for the four genomes that were sequenced in this study. [file 13099_2021_458_MOESM2_ESM.docx]

**Table S2.** List of information for the four genomes that were sequenced in this study.

| Strain | Genome size (bp) | N50 value | L50 value | GC content (%) | Number of contigs | Plasmid info |
| --- | --- | --- | --- | --- | --- | --- |
| CfrFF141 | 5399041 | 729166 | 2 | 51.9 | 88 | RepA_1_pKPC-CAV1321 |
| CfrFF371 | 5293207 | 627142 | 3 | 52.0 | 180 | IncR |
| CfrFF414 | 5307530 | 679759 | 3 | 51.9 | 201 | IncR |
| CfrFF423 | 5297304 | 627142 | 3 | 52.0 | 216 | IncR |
