# Supplementary material for: Comparative genomic characterization of multidrug-resistant Citrobacter spp. strains in Fennec fox imported to China
Source: Gut Pathog. 2021 Oct 13;13:59. doi: 10.1186/s13099-021-00458-w (PMC8513245; doi:10.1186/s13099-021-00458-w)
Supplement: Supplementary file 3 — Additional file 3: Table S2. List of strains and genome sequences that were used in this study. [file 13099_2021_458_MOESM3_ESM.docx]

**Table S1.** List of strains and genome sequences that were used in this study.

| Strain | Species | BioSample | Assembly | Location | Isolation type | Create Date |
| --- | --- | --- | --- | --- | --- | --- |
| Ballerup 7851 | Citrobacter freundii | SAMEA2272125 | GCA_000208765.1 | not collected | environmental | 2011 |
| MTCC 1658 | Citrobacter freundii | SAMN02469678 | GCA_000312465.1 | USA | environmental | 2012 |
| GTC 09479 | Citrobacter freundii | SAMN02469850 | GCA_000342325.1 | Japan | clinical | 2013 |
| GTC 09629 | Citrobacter freundii | SAMN02469849 | GCA_000388155.1 | not collected | environmental | 2013 |
| UCI 31 | Citrobacter freundii | SAMN02356611 | GCA_000521965.1 | USA | clinical | 2014 |
| RLS1 | Citrobacter freundii | SAMN02951916 | GCA_000582615.1 | not collected | environmental | 2014 |
| MGH 56 | Citrobacter freundii | SAMN02581388 | GCA_000692115.1 | USA | clinical | 2014 |
| 5-172-05_S1_C1 | Citrobacter freundii | SAMN02680232 | GCA_000714305.1 | Tanzania | clinical | 2014 |
| ATCC 8090 | Citrobacter freundii | SAMN02742758 | GCA_000734905.1 | Netherlands | environmental | 2014 |
| NBRC 12681 | Citrobacter freundii | SAMD00018684 | GCA_000759735.1 | not collected | environmental | 2014 |
| NBRC 105721 | Citrobacter freundii | SAMD00018685 | GCA_000759755.1 | Belgium | environmental | 2014 |
| FDAARGOS_73 | Citrobacter freundii | SAMN02934525 | GCA_000783755.2 | USA | clinical | 2018 |
| FDAARGOS_61 | Citrobacter freundii | SAMN02934510 | GCA_000783995.2 | USA | clinical | 2018 |
| GTA-CB01 | Citrobacter freundii | SAMN03025290 | GCA_000786265.1 | Canada | environmental | 2014 |
| GTA-CB04 | Citrobacter freundii | SAMN03025291 | GCA_000786275.1 | Canada | environmental | 2014 |
| CIP 55.13 | Citrobacter freundii | SAMEA3099656 | GCA_000826205.1 | not collected | environmental | 2015 |
| MRSN 11938 | Citrobacter freundii | SAMN03316839 | GCA_000937455.2 | USA | clinical | 2015 |
| MRSN 12115 | Citrobacter freundii | SAMN03316840 | GCA_000937505.2 | USA | clinical | 2015 |
| GTA-817-RBA-P2 | Citrobacter freundii | SAMN03437259 | GCA_000972645.1 | Canada | environmental | 2015 |
| SA79 | Citrobacter freundii | SAMN03325972 | GCA_000982845.1 | United Kingdom | clinical | 2015 |
| CAV1321 | Citrobacter freundii | SAMN03733741 | GCA_001022155.1 | USA | clinical | 2015 |
| CAV1741 | Citrobacter freundii | SAMN03733754 | GCA_001022275.1 | USA | clinical | 2015 |
| GN02600 | Citrobacter freundii | SAMN03732718 | GCA_001022685.1 | USA | clinical | 2015 |
| 537_CKOS | Citrobacter freundii | SAMN03197733 | GCA_001055295.1 | USA | clinical | 2015 |
| 538_CKOS | Citrobacter freundii | SAMN03197734 | GCA_001055315.1 | USA | clinical | 2015 |
| 554_CKOS | Citrobacter freundii | SAMN03197753 | GCA_001057215.1 | USA | clinical | 2015 |
| 804_CKOS | Citrobacter freundii | SAMN03198018 | GCA_001058505.1 | USA | clinical | 2015 |
| 874_CKOS | Citrobacter freundii | SAMN03198081 | GCA_001058675.1 | USA | clinical | 2015 |
| 873_CKOS | Citrobacter freundii | SAMN03198080 | GCA_001059255.1 | USA | clinical | 2015 |
| 989_CKOS | Citrobacter freundii | SAMN03198210 | GCA_001059745.1 | USA | clinical | 2015 |
| 1175_SENT | Citrobacter freundii | SAMN03197132 | GCA_001062165.1 | USA | clinical | 2015 |
| 641_SENT | Citrobacter freundii | SAMN03197842 | GCA_001065805.1 | USA | clinical | 2015 |
| 980_SSON | Citrobacter freundii | SAMN03198201 | GCA_001067775.1 | USA | clinical | 2015 |
| WCHCF65 | Citrobacter freundii | SAMN03975633 | GCA_001273815.1 | China | environmental | 2015 |
| ST1 | Citrobacter freundii | SAMN04038590 | GCA_001306025.1 | China | environmental | 2015 |
| ST62:944112508 | Citrobacter freundii | SAMN04011435 | GCA_001316675.1 | South Africa | clinical | 2015 |
| ST63:944526466 | Citrobacter freundii | SAMN04011451 | GCA_001317135.2 | South Africa | clinical | 2016 |
| 953086287 | Citrobacter freundii | SAMN04011453 | GCA_001317155.2 | South Africa | clinical | 2016 |
| ST2 | Citrobacter freundii | SAMN04088747 | GCA_001411885.1 | China | environmental | 2015 |
| RU2 | Citrobacter freundii | SAMN02872722 | GCA_001412715.1 | USA | clinical | 2015 |
| RU2 LB26 | Citrobacter freundii | SAMN02872766 | GCA_001412725.1 | USA | clinical | 2015 |
| RU2 LB29 | Citrobacter freundii | SAMN02872769 | GCA_001412735.1 | USA | clinical | 2015 |
| RU2 LB36 | Citrobacter freundii | SAMN02872776 | GCA_001412755.1 | USA | clinical | 2015 |
| RU2 BHI14 | Citrobacter freundii | SAMN02872790 | GCA_001412795.1 | USA | clinical | 2015 |
| RU2 BHI16 | Citrobacter freundii | SAMN02872792 | GCA_001412815.1 | USA | clinical | 2015 |
| RU2 BHI20 | Citrobacter freundii | SAMN02872796 | GCA_001412825.1 | USA | clinical | 2015 |
| RU2 BHI23 | Citrobacter freundii | SAMN02872799 | GCA_001412855.1 | USA | clinical | 2015 |
| RU2 BHI24 | Citrobacter freundii | SAMN02872800 | GCA_001412875.1 | USA | clinical | 2015 |
| RU2 LB25 | Citrobacter freundii | SAMN02872765 | GCA_001413035.1 | USA | clinical | 2015 |
| RU2 LB27 | Citrobacter freundii | SAMN02872767 | GCA_001413045.1 | USA | clinical | 2015 |
| RU2 LB28 | Citrobacter freundii | SAMN02872768 | GCA_001413075.1 | USA | clinical | 2015 |
| RU2 LB30 | Citrobacter freundii | SAMN02872770 | GCA_001413095.1 | USA | clinical | 2015 |
| RU2 LB32 | Citrobacter freundii | SAMN02872772 | GCA_001413115.1 | USA | clinical | 2015 |
| RU2 LB31 | Citrobacter freundii | SAMN02872771 | GCA_001413125.1 | USA | clinical | 2015 |
| RU2 LB33 | Citrobacter freundii | SAMN02872773 | GCA_001413155.1 | USA | clinical | 2015 |
| RU2 LB34 | Citrobacter freundii | SAMN02872774 | GCA_001413165.1 | USA | clinical | 2015 |
| RU2 LB35 | Citrobacter freundii | SAMN02872775 | GCA_001413195.1 | USA | clinical | 2015 |
| RU2 BHI13 | Citrobacter freundii | SAMN02872789 | GCA_001413215.1 | USA | clinical | 2015 |
| RU2 BHI15 | Citrobacter freundii | SAMN02872791 | GCA_001413235.1 | USA | clinical | 2015 |
| RU2 BHI17 | Citrobacter freundii | SAMN02872793 | GCA_001413255.1 | USA | clinical | 2015 |
| RU2 BHI18 | Citrobacter freundii | SAMN02872794 | GCA_001413275.1 | USA | clinical | 2015 |
| RU2 BHI19 | Citrobacter freundii | SAMN02872795 | GCA_001413295.1 | USA | clinical | 2015 |
| RU2 BHI21 | Citrobacter freundii | SAMN02872797 | GCA_001413315.1 | USA | clinical | 2015 |
| RU2 BHI22 | Citrobacter freundii | SAMN02872798 | GCA_001413335.1 | USA | clinical | 2015 |
| AMA 754 | Citrobacter freundii | SAMN04124434 | GCA_001482545.1 | Denmark | clinical | 2015 |
| AMA 948 | Citrobacter freundii | SAMN04124438 | GCA_001482575.1 | Denmark | clinical | 2015 |
| GED7749C | Citrobacter freundii | SAMN03842472 | GCA_001546285.1 | not collected | clinical | 2016 |
| A47 | Citrobacter freundii | SAMN04246388 | GCA_001689745.1 | Canada | environmental | 2016 |
| B38 | Citrobacter freundii | SAMN05464624 | GCA_001702455.1 | China | clinical | 2016 |
| SL151 | Citrobacter freundii | SAMN05570461 | GCA_001718055.1 | Sierra Leone | clinical | 2016 |
| CF6_ST18 | Citrobacter freundii | SAMN05441405 | GCA_001880795.1 | Spain | clinical | 2016 |
| CF8_ST22 | Citrobacter freundii | SAMN05441430 | GCA_001880825.1 | Spain | clinical | 2016 |
| CF10_ST11 | Citrobacter freundii | SAMN05447746 | GCA_001880835.1 | Spain | clinical | 2016 |
| CF7_ST91 | Citrobacter freundii | SAMN05441416 | GCA_001880845.1 | Spain | clinical | 2016 |
| CF_12_ST_92 | Citrobacter freundii | SAMN05447748 | GCA_001880865.1 | Spain | clinical | 2016 |
| CF_13_ST_93 | Citrobacter freundii | SAMN05447751 | GCA_001880905.1 | Spain | clinical | 2016 |
| CF_15_ST_94 | Citrobacter freundii | SAMN05447777 | GCA_001880915.1 | Spain | clinical | 2016 |
| CF_11_ST_64 | Citrobacter freundii | SAMN05447747 | GCA_001880945.1 | Spain | clinical | 2016 |
| CF_14_ST_18 | Citrobacter freundii | SAMN05447752 | GCA_001880955.1 | Spain | clinical | 2016 |
| BD | Citrobacter freundii | SAMN06158407 | GCA_001922445.1 | China | environmental | 2016 |
| SCC4 | Citrobacter freundii | SAMN06215934 | GCA_001952715.2 | China | environmental | 2017 |
| BF-6 | Citrobacter freundii | SAMN06444903 | GCA_002025225.1 | China | environmental | 2017 |
| FDAARGOS_253 | Citrobacter freundii | SAMN04875579 | GCA_002073755.2 | USA | clinical | 2018 |
| ATCC 51113 | Citrobacter freundii | SAMN06219870 | GCA_002075345.1 | France | environmental | 2017 |
| AK-8 | Citrobacter freundii | SAMN06660612 | GCA_002114305.1 | India | clinical | 2017 |
| MGH142 | Citrobacter freundii | SAMN04521891 | GCA_002151735.1 | USA | clinical | 2017 |
| MGH145 | Citrobacter freundii | SAMN04521894 | GCA_002151755.1 | USA | clinical | 2017 |
| MGH141 | Citrobacter freundii | SAMN04521890 | GCA_002151775.1 | USA | clinical | 2017 |
| MGH146 | Citrobacter freundii | SAMN04521895 | GCA_002151785.1 | USA | clinical | 2017 |
| MGH152 | Citrobacter freundii | SAMN04521901 | GCA_002151815.1 | USA | clinical | 2017 |
| CRK0001 | Citrobacter freundii | SAMN06812673 | GCA_002185305.2 | USA | clinical | 2018 |
| D36-1 | Citrobacter freundii | SAMN06649984 | GCA_002189125.1 | China | environmental | 2017 |
| DPB_2 | Citrobacter freundii | SAMN06009454 | GCA_002192675.1 | USA | environmental | 2017 |
| FDAARGOS_290 | Citrobacter freundii | SAMN06173303 | GCA_002208845.2 | USA | clinical | 2018 |
| 705SK3 | Citrobacter freundii | SAMN07260758 | GCA_002211705.1 | Switzerland | environmental | 2017 |
| 18-1 | Citrobacter freundii | SAMN07280739 | GCA_002215385.1 | China | environmental | 2017 |
| C1710 | Citrobacter freundii | SAMN06820760 | GCA_002239605.1 | China | environmental | 2017 |
| C191 | Citrobacter freundii | SAMN06820765 | GCA_002252025.1 | China | environmental | 2017 |
| 181 | Citrobacter freundii | SAMN06820763 | GCA_002252065.1 | China | environmental | 2017 |
| c196 | Citrobacter freundii | SAMN06820767 | GCA_002252125.1 | China | environmental | 2017 |
| CF003 | Citrobacter freundii | SAMN06106877 | GCA_002417525.1 | South Africa | clinical | 2017 |
| CF004 | Citrobacter freundii | SAMN06106878 | GCA_002417535.1 | South Africa | clinical | 2017 |
| CWH001 | Citrobacter freundii | SAMN07736518 | GCA_002738435.1 | China | clinical | 2017 |
| CRCB-101 | Citrobacter freundii | SAMN07944242 | GCA_002786865.1 | South Korea | clinical | 2017 |
| AR_0022 | Citrobacter freundii | SAMN04014863 | GCA_002796505.1 | not collected | clinical | 2017 |
| YDC691 | Citrobacter freundii | SAMN07974441 | GCA_002863945.1 | USA | clinical | 2018 |
| YDC692-2 | Citrobacter freundii | SAMN07974440 | GCA_002864025.1 | USA | clinical | 2018 |
| UMB1094 | Citrobacter freundii | SAMN07511396 | GCA_002871775.1 | USA | clinical | 2018 |
| S1280 | Citrobacter freundii | SAMN08395897 | GCA_002918455.1 | not collected | environmental | 2018 |
| S1283 | Citrobacter freundii | SAMN08395900 | GCA_002918465.1 | not collected | environmental | 2018 |
| S1278 | Citrobacter freundii | SAMN08395895 | GCA_002918495.1 | not collected | environmental | 2018 |
| S649 | Citrobacter freundii | SAMN08395889 | GCA_002918575.1 | not collected | environmental | 2018 |
| S1282 | Citrobacter freundii | SAMN08395899 | GCA_002919425.1 | not collected | environmental | 2018 |
| S1279 | Citrobacter freundii | SAMN08395896 | GCA_002919455.1 | not collected | environmental | 2018 |
| S1281 | Citrobacter freundii | SAMN08395898 | GCA_002919485.1 | not collected | environmental | 2018 |
| HH1 | Citrobacter freundii | SAMN07318042 | GCA_002923765.1 | Canada | environmental | 2018 |
| CA-26 | Citrobacter freundii | SAMN06234158 | GCA_002939255.1 | Bolivia | environmental | 2018 |
| 236-17-2 | Citrobacter freundii | SAMN08426773 | GCA_003015305.1 | Germany | environmental | 2018 |
| B9-C2 | Citrobacter freundii | SAMN08631944 | GCA_003019835.1 | Malaysia | clinical | 2018 |
| 164 | Citrobacter freundii | SAMN08942770 | GCA_003070705.1 | Argentina | clinical | 2018 |
| MH17-012N | Citrobacter freundii | SAMD00112927 | GCA_003114915.2 | Vietnam | clinical | 2020 |
| MH16-522D | Citrobacter freundii | SAMD00112928 | GCA_003114935.2 | Vietnam | clinical | 2020 |
| TUM1507 | Citrobacter freundii | SAMD00115687 | GCA_003175795.1 | not collected | clinical | 2018 |
| WUSM_CF_61 | Citrobacter freundii | SAMN09266862 | GCA_003195445.1 | USA | clinical | 2018 |
| L6 | Citrobacter freundii | SAMN07206908 | GCA_003255895.1 | South Korea | environmental | 2018 |
| 1-RC-17-03941 | Citrobacter freundii | SAMN09694014 | GCA_003362775.1 | USA | clinical | 2018 |
| TV1575CF | Citrobacter freundii | SAMN09736005 | GCA_003363295.1 | Brazil | clinical | 2018 |
| CRE89 | Citrobacter freundii | SAMN08623837 | GCA_003400395.1 | USA | clinical | 2018 |
| CRE20 | Citrobacter freundii | SAMN08623795 | GCA_003401455.1 | USA | clinical | 2018 |
| FDA-CDC-AR_0116 | Citrobacter freundii | SAMN04014957 | GCA_003571565.1 | not collected | clinical | 2018 |
| UMH19 | Citrobacter freundii | SAMN07729551 | GCA_003665535.1 | USA | clinical | 2018 |
| UMH18 | Citrobacter freundii | SAMN07729550 | GCA_003665555.1 | USA | clinical | 2018 |
| UMH17 | Citrobacter freundii | SAMN07729549 | GCA_003665575.1 | USA | clinical | 2018 |
| UMH15 | Citrobacter freundii | SAMN07729547 | GCA_003665595.1 | USA | clinical | 2018 |
| UMH14 | Citrobacter freundii | SAMN07729546 | GCA_003665615.1 | USA | clinical | 2018 |
| UMH13 | Citrobacter freundii | SAMN07729545 | GCA_003665635.1 | USA | clinical | 2018 |
| HM38 | Citrobacter freundii | SAMN07729552 | GCA_003665655.1 | USA | clinical | 2018 |
| UMH16 | Citrobacter freundii | SAMN07729548 | GCA_003665675.1 | USA | clinical | 2018 |
| 1019002 | Citrobacter freundii | SAMN10252246 | GCA_003687835.1 | Germany | environmental | 2018 |
| FDAARGOS_550 | Citrobacter freundii | SAMN10163243 | GCA_003812325.1 | not collected | clinical | 2018 |
| FDAARGOS_549 | Citrobacter freundii | SAMN10163242 | GCA_003812345.1 | not collected | clinical | 2018 |
| CCUG 30791 | Citrobacter freundii | SAMN10440282 | GCA_003818115.1 | USA | environmental | 2018 |
| CF_324 | Citrobacter freundii | SAMN10249196 | GCA_003937345.1 | Pakistan | environmental | 2018 |
| 064C1 | Citrobacter freundii | SAMN10644710 | GCA_004004645.1 | France | environmental | 2019 |
| 064B9 | Citrobacter freundii | SAMN10644708 | GCA_004004705.1 | France | environmental | 2019 |
| 064B7 | Citrobacter freundii | SAMN10644706 | GCA_004004715.1 | France | environmental | 2019 |
| 109A8 | Citrobacter freundii | SAMN10644703 | GCA_004004805.1 | France | environmental | 2019 |
| 122E9 | Citrobacter freundii | SAMN10644693 | GCA_004004895.1 | France | clinical | 2019 |
| 116E5 | Citrobacter freundii | SAMN10644688 | GCA_004004965.1 | France | clinical | 2019 |
| 106F1 | Citrobacter freundii | SAMN10644678 | GCA_004005035.1 | France | clinical | 2019 |
| 102B9 | Citrobacter freundii | SAMN10644675 | GCA_004005085.1 | France | clinical | 2019 |
| 064B8 | Citrobacter freundii | SAMN10644707 | GCA_004005165.1 | France | environmental | 2019 |
| 109A5 | Citrobacter freundii | SAMN10644700 | GCA_004005235.1 | France | environmental | 2019 |
| 109A3 | Citrobacter freundii | SAMN10644698 | GCA_004005255.1 | France | environmental | 2019 |
| 106F2 | Citrobacter freundii | SAMN10644679 | GCA_004005575.1 | France | clinical | 2019 |
| 122E5 | Citrobacter freundii | SAMN10644671 | GCA_004005615.1 | France | clinical | 2019 |
| GEO_48_Eff_C | Citrobacter freundii | SAMN09289757 | GCA_004023995.1 | USA | environmental | 2019 |
| GEO_21_Eff_A | Citrobacter freundii | SAMN09289739 | GCA_004024365.1 | USA | environmental | 2019 |
| GEO_16_Eff_A | Citrobacter freundii | SAMN09289737 | GCA_004024405.1 | USA | environmental | 2019 |
| R17 | Citrobacter freundii | SAMN10754625 | GCA_004103775.1 | China | environmental | 2019 |
| 071G10 | Citrobacter freundii | SAMN10840491 | GCA_004145665.1 | France | clinical | 2019 |
| ICR003215 | Citrobacter freundii | SAMN10847473 | GCA_004146055.1 | France | clinical | 2019 |
| 116E2 | Citrobacter freundii | SAMN10644685 | GCA_004152635.1 | France | clinical | 2019 |
| 109A10 | Citrobacter freundii | SAMN10644682 | GCA_004153245.1 | France | clinical | 2019 |
| ICR003203 | Citrobacter freundii | SAMN10847461 | GCA_004181935.1 | France | clinical | 2019 |
| IFO 13545 | Citrobacter freundii | SAMD00143536 | GCA_004305705.1 | not collected | environmental | 2019 |
| ACFMW | Citrobacter freundii | SAMN10794296 | GCA_004327735.1 | USA | clinical | 2019 |
| 2016WA-SCV | Citrobacter freundii | SAMN10976862 | GCA_004327825.1 | USA | clinical | 2019 |
| 2016WA-NCV | Citrobacter freundii | SAMN10976863 | GCA_004327835.1 | USA | clinical | 2019 |
| HH10 | Citrobacter freundii | SAMN10437855 | GCA_004331445.1 | Canada | environmental | 2019 |
| HH8 | Citrobacter freundii | SAMN10437853 | GCA_004331535.1 | Canada | environmental | 2019 |
| HH7 | Citrobacter freundii | SAMN10437852 | GCA_004331545.1 | Canada | environmental | 2019 |
| HH11 | Citrobacter freundii | SAMN10437856 | GCA_004331565.1 | Canada | environmental | 2019 |
| HH5 | Citrobacter freundii | SAMN10437850 | GCA_004331575.1 | Canada | environmental | 2019 |
| HH6 | Citrobacter freundii | SAMN10437851 | GCA_004331585.1 | Canada | environmental | 2019 |
| HH9 | Citrobacter freundii | SAMN10437854 | GCA_004331635.1 | Canada | environmental | 2019 |
| CAV1857 | Citrobacter freundii | SAMN11055879 | GCA_004344985.1 | USA | environmental | 2019 |
| MGF016 | Citrobacter freundii | SAMN11366429 | GCA_004795555.1 | Malaysia | clinical | 2019 |
| P079F I | Citrobacter freundii | SAMN09204089 | GCA_004801105.1 | United Kingdom | clinical | 2019 |
| P106E PI | Citrobacter freundii | SAMN09204104 | GCA_004801115.1 | United Kingdom | clinical | 2019 |
| 2016WA-REV | Citrobacter freundii | SAMN10976864 | GCA_005217825.1 | USA | clinical | 2019 |
| R47 | Citrobacter freundii | SAMN11845725 | GCA_005960425.1 | China | environmental | 2019 |
| 163RA | Citrobacter freundii | SAMN12025733 | GCA_006788685.1 | USA | environmental | 2019 |
| 92RB2 | Citrobacter freundii | SAMN12025786 | GCA_006788725.1 | USA | environmental | 2019 |
| 94RA | Citrobacter freundii | SAMN12024903 | GCA_006788735.1 | USA | environmental | 2019 |
| 57RB | Citrobacter freundii | SAMN12024776 | GCA_006788745.1 | USA | environmental | 2019 |
| 51R | Citrobacter freundii | SAMN12025567 | GCA_006788755.1 | USA | environmental | 2019 |
| 98R | Citrobacter freundii | SAMN12024695 | GCA_006788775.1 | USA | environmental | 2019 |
| 154R | Citrobacter freundii | SAMN12025732 | GCA_006788825.1 | USA | environmental | 2019 |
| 166R | Citrobacter freundii | SAMN12025566 | GCA_006788835.1 | USA | environmental | 2019 |
| 191T | Citrobacter freundii | SAMN12025568 | GCA_006788865.1 | USA | environmental | 2019 |
| EELTKB284 | Citrobacter freundii | SAMN11232674 | GCA_006937845.1 | Estonia | clinical | 2019 |
| INSACf32145 | Citrobacter freundii | SAMN12219522 | GCA_007004285.1 | Portugal | clinical | 2019 |
| KCJ3K347 | Citrobacter freundii | SAMN12628157 | GCA_008180565.1 | USA | clinical | 2019 |
| C8 | Citrobacter freundii | SAMN12560195 | GCA_008364635.1 | Germany | environmental | 2019 |
| C7 | Citrobacter freundii | SAMN12560194 | GCA_008364705.1 | Germany | environmental | 2019 |
| BA18 | Citrobacter freundii | SAMN09744871 | GCA_008373795.1 | Portugal | environmental | 2019 |
| CC12 | Citrobacter freundii | SAMN09862364 | GCA_008502135.1 | Nigeria | environmental | 2019 |
| FDAARGOS_616 | Citrobacter freundii | SAMN11056331 | GCA_008693645.1 | USA | clinical | 2019 |
| C50 | Citrobacter freundii | SAMN12289284 | GCA_008931485.1 | Australia | clinical | 2019 |
| E33 | Citrobacter freundii | SAMN12289346 | GCA_008931505.1 | Australia | environmental | 2019 |
| E11 | Citrobacter freundii | SAMN12289395 | GCA_008931625.1 | Australia | environmental | 2019 |
| E51 | Citrobacter freundii | SAMN12289332 | GCA_008931685.1 | Australia | environmental | 2019 |
| MY49 | Citrobacter freundii | SAMN13064487 | GCA_009647535.1 | China | environmental | 2019 |
| MiY-A | Citrobacter freundii | SAMN10574724 | GCA_009648935.1 | USA | environmental | 2019 |
| 154 | Citrobacter freundii | SAMN11287982 | GCA_009664025.1 | Spain | clinical | 2014 |
| 565 | Citrobacter freundii | SAMN11288321 | GCA_009664045.1 | Spain | clinical | 2019 |
| 680 | Citrobacter freundii | SAMN11288343 | GCA_009664065.1 | Spain | clinical | 2019 |
| SC105 | Citrobacter freundii | SAMN11928082 | GCA_009821375.1 | not collected | environmental | 2019 |
| JS97 | Citrobacter freundii | SAMN11928074 | GCA_009821535.1 | not collected | environmental | 2019 |
| SC96 | Citrobacter freundii | SAMN11928073 | GCA_009821555.1 | not collected | environmental | 2019 |
| LDL3-3 | Citrobacter freundii | SAMN13669122 | GCA_009856695.1 | China | environmental | 2020 |
| BOX-5 | Citrobacter freundii | SAMN13623934 | GCA_009856875.1 | France | environmental | 2020 |
| L75 | Citrobacter freundii | SAMN13282750 | GCA_009857035.1 | China | clinical | 2020 |
| 2580 | Citrobacter freundii | SAMN13830004 | GCA_009907085.1 | Nigeria | clinical | 2020 |
| HK1 | Citrobacter freundii | SAMN12096240 | GCA_009910785.1 | South Korea | environmental | 2020 |
| S169 | Citrobacter freundii | SAMN10613361 | GCA_009939625.1 | South Korea | environmental | 2020 |
| UL-CPE-01 | Citrobacter freundii | SAMN13927060 | GCA_010119415.1 | Ireland | environmental | 2020 |
| CR16 | Citrobacter freundii | SAMN10171331 | GCA_010279875.1 | Portugal | environmental | 2020 |
| 62 | Citrobacter freundii | SAMN13951913 | GCA_010365585.1 | Switzerland | environmental | 2020 |
| AS012461 | Citrobacter freundii | SAMN12250780 | GCA_010590395.1 | USA | clinical | 2020 |
| AS012430 | Citrobacter freundii | SAMN12250749 | GCA_010590925.1 | USA | clinical | 2020 |
| AS012402 | Citrobacter freundii | SAMN12250721 | GCA_010592375.1 | USA | clinical | 2020 |
| AS012330 | Citrobacter freundii | SAMN12250649 | GCA_010597685.1 | USA | clinical | 2020 |
| L91 | Citrobacter freundii | SAMN10743647 | GCA_011022025.1 | China | clinical | 2020 |
| CFM_17 | Citrobacter freundii | SAMN14120105 | GCA_011030065.1 | Lebanon | clinical | 2020 |
| CFM_67 | Citrobacter freundii | SAMN14120186 | GCA_011030295.1 | Lebanon | clinical | 2020 |
| CFM_69 | Citrobacter freundii | SAMN14120187 | GCA_011030315.1 | Lebanon | clinical | 2020 |
| ATCC 8090 | Citrobacter freundii | SAMN14128751 | GCA_011064845.1 | not collected | environmental | 2020 |
| Pc211 | Citrobacter freundii | SAMN14123591 | GCA_011065535.1 | Ecuador | environmental | 2020 |
| Pc112 | Citrobacter freundii | SAMN14123592 | GCA_011065545.1 | Ecuador | environmental | 2020 |
| Pc111 | Citrobacter freundii | SAMN14123590 | GCA_011065655.1 | Ecuador | environmental | 2020 |
| 175G8 | Citrobacter freundii | SAMN12289071 | GCA_011392435.1 | France | clinical | 2020 |
| UFMG-H9 | Citrobacter freundii | SAMN14470508 | GCA_012102405.1 | Brazil | environmental | 2020 |
| RIT669 | Citrobacter freundii | SAMN14521490 | GCA_012641175.1 | USA | environmental | 2020 |
| CB00017 | Citrobacter freundii | SAMN14265977 | GCA_012952645.1 | USA | clinical | 2020 |
| CB00028 | Citrobacter freundii | SAMN14265978 | GCA_012952665.1 | USA | clinical | 2020 |
| SCAID PHRX1-2019 | Citrobacter freundii | SAMN14653284 | GCA_012955545.1 | Kazakhstan | clinical | 2020 |
| SCAID URN1-2019 | Citrobacter freundii | SAMN14653268 | GCA_012955585.1 | Kazakhstan | clinical | 2020 |
| C2-03 | Citrobacter freundii | SAMN11394775 | GCA_013168955.1 | Colombia | clinical | 2020 |
| C1-185 | Citrobacter freundii | SAMN11394728 | GCA_013168975.1 | Colombia | clinical | 2020 |
| C2-132 | Citrobacter freundii | SAMN07291392 | GCA_013170035.1 | Colombia | clinical | 2020 |
| C2-135 | Citrobacter freundii | SAMN07291393 | GCA_013170055.1 | Colombia | clinical | 2020 |
| CRN 21 | Citrobacter freundii | SAMN14531572 | GCA_013266895.1 | India | environmental | 2020 |
| FDAARGOS_638 | Citrobacter freundii | SAMN11056353 | GCA_013267145.1 | USA | clinical | 2020 |
| D9 | Citrobacter freundii | SAMEA103915302 | GCA_900169625.1 | not collected | environmental | 2017 |
| D8 | Citrobacter freundii | SAMEA103915301 | GCA_900169695.1 | not collected | environmental | 2017 |
| NCTC6266 | Citrobacter freundii | SAMEA4552893 | GCA_900446875.1 | not collected | environmental | 2018 |
| NCTC8165 | Citrobacter freundii | SAMEA26392918 | GCA_900446885.1 | United Kingdom | environmental | 2018 |
| NCTC13708 | Citrobacter freundii | SAMEA3714888 | GCA_900446905.1 | USA | environmental | 2018 |
| NCTC13639 | Citrobacter freundii | SAMEA2742611 | GCA_900460895.1 | not collected | environmental | 2018 |
| NCTC13630 | Citrobacter freundii | SAMEA2742609 | GCA_900460935.1 | not collected | environmental | 2018 |
| NCTC6267 | Citrobacter freundii | SAMEA2665120 | GCA_900460945.1 | not collected | environmental | 2018 |
| E2614 | Citrobacter freundii | SAMEA4830817 | GCA_900520335.1 | not collected | environmental | 2019 |
| U2785 | Citrobacter freundii | SAMEA4830818 | GCA_900520375.1 | not collected | environmental | 2019 |
| NCTC13709 | Citrobacter freundii | SAMEA103980411 | GCA_900638065.1 | USA | environmental | 2018 |
| 30_P_CF | Citrobacter freundii | SAMEA5578849 | GCA_901456225.1 | Germany | clinical | 2019 |
| 32_P_CF | Citrobacter freundii | SAMEA5578852 | GCA_901456255.1 | Germany | clinical | 2019 |
| 27_P_CF | Citrobacter freundii | SAMEA5578841 | GCA_901456285.1 | Germany | clinical | 2019 |
| 29_P_CF | Citrobacter freundii | SAMEA5578845 | GCA_901456305.1 | Germany | clinical | 2019 |
| 4928STDY7071459 | Citrobacter freundii | SAMEA104567567 | GCA_902160715.1 | United Kingdom | clinical | 2019 |
| 4928STDY7071491 | Citrobacter freundii | SAMEA104567598 | GCA_902160855.1 | United Kingdom | clinical | 2019 |
| 4928STDY7071493 | Citrobacter freundii | SAMEA104567600 | GCA_902160915.1 | United Kingdom | clinical | 2019 |
| 4928STDY7071492 | Citrobacter freundii | SAMEA104567599 | GCA_902160945.1 | United Kingdom | clinical | 2019 |
| 4928STDY7071494 | Citrobacter freundii | SAMEA104567601 | GCA_902160995.1 | United Kingdom | clinical | 2019 |
| 4928STDY7387836 | Citrobacter freundii | SAMEA104694472 | GCA_902165615.1 | United Kingdom | clinical | 2019 |
| 4928STDY7071646 | Citrobacter freundii | SAMEA104567751 | GCA_902166445.1 | United Kingdom | clinical | 2019 |
| MGYG-HGUT-00108 | Citrobacter freundii | SAMEA5849609 | GCA_902363265.1 | not collected | environmental | 2019 |
| MGYG-HGUT-02495 | Citrobacter freundii | SAMEA5852000 | GCA_902387635.1 | USA | environmental | 2019 |
| PBIO701 | Citrobacter freundii | SAMEA6574524 | GCA_902807175.1 | Rwanda | environmental | 2020 |
| PBIO1938 | Citrobacter freundii | SAMEA6574534 | GCA_902807235.1 | Rwanda | environmental | 2020 |
| PBIO1950 | Citrobacter freundii | SAMEA6574546 | GCA_902807305.1 | Rwanda | environmental | 2020 |
| CfrFF141 | Citrobacter cronae | SAMN15770403 | GCF_014230205.1 | Sudan | animal | 2018 |
| CfrFF371 | Citrobacter braakii | SAMN15770404 | [GCA_014230185.1](https://www.ncbi.nlm.nih.gov/assembly/GCA_014230185.1) | Sudan | animal | 2018 |
| CfrFF414 | Citrobacter braakii | SAMN15770405 | GCA_014230155.1 | Sudan | animal | 2018 |
| CfrFF423 | Citrobacter braakii | SAMN15770406 | GCA_014230105.1 | Sudan | animal | 2018 |
